# Supplementary material for: Mapping the evolution of fertility support policies in China: A content and instrumental analysis
Source: PLoS One. 2025 Oct 9;20(10):e0332137. doi: 10.1371/journal.pone.0332137 (PMC12510515; doi:10.1371/journal.pone.0332137)
Supplement: S1 Appendix — (ZIP) [file pone.0332137.s001.zip › S1 Appendix. 226 original policy documents/175-中国人民解放军计划生育条例(2003)(FBM-CLI-7-229227).doc]

中国人民解放军计划生育条例(2003)

**发布部门：** [中央军委](https://www.pkulaw.com/law/chl?Aggs.IssueDepartment=b01)

**发布日期：**2003.04.08

**实施日期：**2003.04.08

**时效性：** [已被修改](https://www.pkulaw.com/law/chl?Aggs.TimelinessDic=03)

**效力级别：** [军事法规](https://www.pkulaw.com/law/chl?Aggs.EffectivenessDic=XQ0901)

**法规类别：** [计划生育管理](https://www.pkulaw.com/law/chl?Aggs.Category=09705)

**修改依据：**[中央军委关于修改《中国人民解放军计划生育条例》的决定(2014)](javascript:TMP(229226,0,'chl'))

中国人民解放军计划生育条例
（中央军委 2003年4月8日）

目录

　　第一章 总则
　　第二章 机构与职责
　　第三章 生育调节
　　第四章 技术服务
　　第五章 优待与奖励
　　第六章 法律责任
　　第七章 附则

　第一章　总则

　　**第一条**　为了加强军队计划生育工作，保障实行计划生育人员的合法权益，根据《[中华人民共和国人口与计划生育法](https://www.pkulaw.com/chl/252f7ce51132230bbdfb.html)》，结合军队实际，制定本条例。

　　**第二条**　军队所有单位必须贯彻执行国家和军队有关计划生育的法律、法规和规章，加强对计划生育工作的领导，健全组织机构，保障必要的工作经费，实行目标管理责任制，依法管理计划生育。

　　**第三条**　军队所有人员应当遵守国家和军队有关计划生育的法律、法规和规章，自觉实行计划生育。

　　**第四条**　军队依法保障军队人员合法生育、免费享受国家规定的基本项目的计划生育技术服务的权利以及法律、法规规定的其他实行计划生育的权益。

　　**第五条**　军队计划生育工作贯彻宣传教育为主、避孕为主、经常性工作为主的方针，坚持与部队全面建设相结合，与官兵成长进步相结合，与建立文明幸福家庭相结合。

　第二章　机构与职责

　　**第六条**　中国人民解放军计划生育领导小组在中央军委领导下，负责军队计划生育工作的组织领导，履行下列职责：
　　（一）依据国家有关法律、法规，组织拟制军队计划生育工作的方针、政策和法规，指导全军开展计划生育工作；
　　（二）审定全军计划生育工作的长远规划和年度计划；
　　（三）审批军师职领导干部人口理论集训计划；
　　（四）定期总结全军计划生育工作，推广计划生育工作经验；
　　（五）组织实施全军性计划生育工作的评比、表彰活动；
　　（六）中央军委赋予的其他职责。

　　**第七条**　中国人民解放军计划生育领导小组办公室承办中国人民解放军计划生育领导小组的日常工作，履行下列职责：
　　（一）拟制全军计划生育工作长远规划和年度计划，并组织实施，监督执行；
　　（二）组织和指导全军计划生育宣传教育和婚育管理；
　　（三）组织军队计划生育科学研究和技术服务；
　　（四）组织军师职领导干部人口理论集训和军级以上单位计划生育干部培训；
　　（五）编报全军计划生育工作经费预算和决算，监督、检查计划生育工作经费的使用管理；
　　（六）制定全军避孕药具需求计划，负责避孕药具的订购、分配和管理；
　　（七）负责全军计划生育工作统计，接待和处理群众来信来访；
　　（八）中国人民解放军计划生育领导小组赋予的其他职责。

　　**第八条**　团级以上单位计划生育领导小组在同级党委领导下，组织领导本单位的计划生育工作，履行下列职责：
　　（一）贯彻执行国家和军队有关法律、法规和规章，建立健全计划生育规章制度，指导所属单位开展计划生育工作；
　　（二）审定本单位计划生育工作规划、计划；
　　（三）定期分析计划生育工作情况，推广计划生育工作经验，督促有关部门履行计划生育工作职责；
　　（四）上级赋予的其他职责。

　　**第九条**　团级以上单位计划生育领导小组办公室承办本单位计划生育领导小组的日常工作，履行下列职责：
　　（一）拟制本单位计划生育工作规划、计划，并组织实施，监督执行；
　　（二）组织开展计划生育宣传教育和婚育管理；
　　（三）组织开展计划生育技术服务；
　　（四）组织领导干部人口理论学习和计划生育干部培训；
　　（五）管理使用计划生育工作经费；
　　（六）编报避孕药具需求计划，管理和发放避孕药具；
　　（七）负责计划生育工作统计，接待和处理群众来信来访；
　　（八）上级赋予的其他职责。

　　**第十条**　营、连计划生育指导小组在同级党委（党支部）领导下组织落实本单位计划生育工作。

　　**第十一条**　各级机关的军务、动员、组织、干部、宣传、纪检、财务、卫生等部门，应当按照职责分工，履行有关计划生育工作的职责，协助和配合计划生育管理部门开展计划生育宣传教育、婚育管理和服务保障工作。

　第三章　生育调节

　　**第十二条**　军队鼓励晚婚晚育，提倡一对夫妻生育一个子女。禁止违法生育。男25周岁以上、女23周岁以上初婚者为晚婚。已婚妇女达到晚婚年龄以后怀孕生育第一个子女为晚育。

　　**第十三条**　女方达到晚婚年龄的育龄夫妻，可自行选择怀孕生育第一个子女的时间，并按照女方户籍所在地的规定办理生育证明或者生育服务证。女方为现役军人的，其单位所在地视为户籍所在地。

　　**第十四条**　符合下列条件之一的育龄夫妻，可以申请生育第二个子女：
　　（一）第一个子女为残疾儿，不能成长为正常劳动力，但医学上认为可以再生育的；
　　（二）再婚夫妻一方再婚前只有一个子女，另一方无子女的；
　　（三）患不孕（育）症，依法收养一个子女后又怀孕的；
　　（四）夫妻双方均为独生子女，符合女方户籍所在省、自治区、直辖市规定的生育第二个子女条件的；
　　（五）夫妻双方均为少数民族，符合女方户籍所在省、自治区、直辖市规定的生育第二个子女条件的；
　　（六）职工配偶是农村居民，符合女方户籍所在省、自治区、直辖市规定的生育第二个子女条件的。符合前款规定条件，申请生育第二个子女的，由夫妻双方分别向所在单位的计划生育管理部门提出申请，按照女方户籍所在地的规定取得生育证明或者生育服务证，并经军级单位计划生育领导小组审核批准，方可生育第二个子女。军级单位计划生育领导小组在接到育龄夫妻生育第二个子女的申请后，应当在60天内予以批复。

　　**第十五条**　再婚家庭双方再婚前累计已有两个子女，申请再生育一个子女的，必须符合女方户籍所在省、自治区、直辖市的规定，并取得女方户籍所在地的生育证明或者生育服务证，经军级单位计划生育领导小组审核同意，报军区级单位计划生育领导小组批准，方可再生育一个子女。

　　**第十六条**　育龄夫妻应当自觉落实计划生育避孕节育措施，接受计划生育技术服务指导。预防和减少非意愿妊娠。

　第四章　技术服务

　　**第十七条**　军队建立由各级医疗卫生机构组成的计划生育技术服务网络，开展婚前保健、孕产期保健和计划生育技术服务，提高出生婴儿健康水平。

　　**第十八条**　各级医疗卫生机构应当按照规定，在其职责范围内，开展人口与计划生育基础知识宣传教育，向育龄夫妻提供计划生育、生殖保健的咨询、指导和技术服务。

　　**第十九条**　军队医疗卫生机构应当加强计划生育、生殖保健科学研究，有条件的，经过批准可以开展新业务、新技术。

　　**第二十条**　军队人员结婚前，男女双方必须到军队或者地方指定的医疗机构进行婚前医学检查，接受婚前卫生指导。

　　**第二十一条**　育龄夫妻享有避孕节育方法的知情选择权和安全保障权。提倡已生育一个子女的育龄夫妻选择以长效为主的避孕措施。

　　**第二十二条**　计划生育技术服务人员应当指导育龄夫妻选择安全、有效、适宜的避孕措施。

　　**第二十三条**　军队医疗卫生机构开展计划生育技术服务必须经军队卫生部门批准。计划生育技术服务人员必须经过相应的技术培训，具备规定的资格。施行计划生育手术必须严格遵守技术规范和相关制度，确保受术者的安全。

　　**第二十四条**　在军队医疗卫生机构施行计划生育手术造成并发症，经军区级以上单位计划生育技术鉴定小组鉴定确认的，军队医疗卫生机构应当给予免费治疗。

　　**第二十五条**　各级计划生育领导小组和卫生部门应当对计划生育技术服务工作进行指导和监督。

　　**第二十六条**　严禁任何单位或者人员采用技术手段对胎儿进行非医学需要的性别鉴定和选择性别的人工终止妊娠。

　第五章　优待与奖励

　　**第二十七条**　军队对实行计划生育的育龄夫妻按照规定给予奖励。

　　**第二十八条**　晚婚的军队人员，除享受国家规定的婚假外，增加晚婚假7天。晚婚假期间工资照发，福利待遇不变。夫妻双方同为军队人员，双方达到晚婚年龄的，双方享受晚婚假；一方达到的，一方享受。夫妻一方为地方人员的，按照地方有关规定执行。

　　**第二十九条**　接受计划生育手术的军队人员，享受国家规定的休假待遇。

　　**第三十条**　女军人、女职工符合晚育规定，自愿终生只生育一个子女的，除按照规定享受3个月产假外，增加产假90天。增加产假期间的工资和福利待遇按照产假的规定执行。男方为军队人员，自愿终生只生育一个子女，夫妻异地居住、享受探亲假的，在女方分娩当年给予护理假15天。

　　**第三十一条**　夫妻自愿终生只生育一个子女的，由夫妻双方分别向所在单位的计划生育管理部门提出申请，经批准后在女方户籍所在地办理《独生子女父母光荣证》。自领证之月起，按照规定领取独生子女父母奖励费。育龄夫妻符合下列条件之一的，也可以申请领取《独生子女父母光荣证》，享受有关待遇：
　　（一）原有两个子女，其中一个子女因故死亡后不再生育的；
　　（二）原有一个子女因故死亡，又生育一个子女后不再生育的。

　　**第三十二条**　按照本条例第十四条的规定生育第二个子女的夫妻，所在单位应当自批准之月起停发独生子女父母奖励费，并收回《独生子女父母光荣证》。

　　**第三十三条**　对在计划生育工作中做出显著成绩的单位和个人，依照《[中国人民解放军纪律条令](https://www.pkulaw.com/chl/5f75f42122f758e3bdfb.html)》的有关规定，给予表彰和奖励。

　第六章　法律责任

　　**第三十四条**　违反本条例规定生育第二个子女的，应当按照国家和女方户籍所在省、自治区、直辖市的规定缴纳社会抚养费，所在单位应当收回其领取的《独生子女父母光荣证》和已发给的全部独生子女父母奖励费，并依照《[中国人民解放军纪律条令](https://www.pkulaw.com/chl/5f75f42122f758e3bdfb.html)》的有关规定给予降职（级）或者降衔（级）以上处分。当事人是军队职工的，按照《军队职工奖惩实施办法》的有关规定给予降职（级）以上处分。
　　违反本条例，生育第三个及第三个以上子女的，加重处分。

　　**第三十五条**　以不正当手段取得计划生育证明、生育第二个及第二个以上子女和非法收养子女的，按违法生育处理。

　　**第三十六条**　未完成计划生育工作目标，出现违法生育的单位，当年不得被评为综合性先进单位；对单位主管领导和直接责任人，依照《[中国人民解放军纪律条令](https://www.pkulaw.com/chl/5f75f42122f758e3bdfb.html)》的有关规定，给予处分。

　　**第三十七条**　有下列行为之一的，依照《[中国人民解放军纪律条令](https://www.pkulaw.com/chl/5f75f42122f758e3bdfb.html)》的有关规定，给予处分；有违法所得的，没收违法所得；构成犯罪的，依法追究刑事责任：
　　（一）违反规定为他人进行胎儿性别鉴定或者选择性别的人工终止妊娠的；
　　（二）不具备国家和军队规定的条件和资格实施计划生育手术的；
　　（三）在计划生育技术服务中违章操作或者延误抢救、诊治，造成严重后果的；
　　（四）为计划外怀孕或者计划外生育者逃避管理提供保护的；
　　（五）非法倒卖避孕药具的；
　　（六）实施假节育手术、进行假医学鉴定、出具假计划生育证明的；
　　（七）其他妨害计划生育工作的。

　第七章　附则

　　**第三十八条**　在军队营区居住或者从业的流动人口的计划生育管理，按照国家和所在省、自治区、直辖市以及军队的有关规定执行。

　　**第三十九条**　军队出国留学人员的计划生育管理，按照国家有关规定执行。

　　**第四十条**　中国人民武装警察部队的计划生育工作，适用本条例。

　　**第四十一条**　各总部、军兵种、军区、武警部队可以根据本条例制定实施细则。

　　**第四十二条**　本条例由中国人民解放军计划生育领导小组负责解释。

　　**第四十三条**　本条例自发布之日起施行。1996年1月5日经中央军委批准，总参谋部、总政治部、总后勤部颁布的《中国人民解放军计划生育条例》即行废止。

© 北大法宝：（[www.pkulaw.com](https://www.pkulaw.com/)) 专业提供法律信息、法学知识和法律软件领域各类解决方案。北大法宝为您提供丰富的参考资料，正式引用法规条文时请与标准文本核对。
欢迎查看所有[产品和服务](https://www.pkulaw.com/helps/2.html)。法宝快讯：[如何快速找到您需要的检索结果？](https://www.pkulaw.com/helps/70.html)[法宝](https://www.pkulaw.com/helps/69.html)V6有何新特色？

扫描二维码阅读原文

**原文链接：**<https://www.pkulaw.com/chl/ee14c7a2abc1d4a9bdfb.html>
